# Fracture Resistance of Primary Zirconia Crowns: An In Vitro Study

**DOI:** 10.3390/children9010077

**Published:** 2022-01-05

**Authors:** AlWaleed Abushanan, Rajashekhara B. Sharanesha, Bader Aljuaid, Tariq Alfaifi, Abdullah Aldurayhim

**Affiliations:** 1 Department of Preventive Dental Sciences, College of Dentistry, Prince Sattam bin Abdulaziz University, Al-Kharj 11942, Saudi Arabia; r.bhari@psau.edu.sa; 2 Dental Intern, College of Dentistry, Prince Sattam bin Abdulaziz University, Al-Kharj 11942, Saudi Arabia; b.k.aljuaid@gmail.com (B.A.); dr.tariq0036@gmail.com (T.A.); dr.abdullaaldrihem@gmail.com (A.A.)

**Keywords:** fracture resistance, Instron, primary teeth, universal testing machine, zirconia crowns

## Abstract

In this study, we evaluated the fracture resistance of three commercially available prefabricated primary zirconia crowns and their correlation with dimensional variance. Methods: a total of 42 zirconia crowns were selected from three companies, (1) NuSmile primary zirconia crowns, (2) Cheng Crowns zirconia, and (3) Sprig EZ crowns. The crowns were divided into two groups based on their location in the oral cavity and further divided into subgroups based on the brand. All of the samples were subjected to fracture tests using a universal testing machine. Results: the mean load observed was highest with Cheng Crowns zirconia anterior crowns (1355 ± 484) and the least load was seen with Sprig EZ anterior crowns with a mean load of 339 ± 94. The mean load observed was highest with Cheng Crowns zirconia posterior crowns (1990 ± 485) followed by NuSmile posterior crowns and the least load was seen with Sprig EZ posterior crowns with a mean load of 661 ± 184. Conclusion: the Cheng crowns showed the highest fracture resistance amongst all three groups. Overall, the zirconia crowns (anterior and posterior) tested showed optimum mechanical properties to withstand the masticatory forces.

## 1. Introduction

Early childhood caries (ECC) is the most common childhood disease and is often accompanied by serious comorbidities affecting the quality of life of children, their families, the community, and the health care system [1]. Various materials have been used to extensively treat carious primary teeth, such as amalgam, glass ionomer, composite resin, stainless-steel crowns (SSC), and, recently, zirconia crowns with varying success rates [2]. For nearly 70 years, SSC have been used to restore carious primary molars and have been considered as the gold standard due to their relatively simple procedure, durability, and low cost [3]. However, despite the advantages of SSC, parents are often dissatisfied with their esthetic properties [4]. Primary zirconia crowns were introduced to the market over 10 years ago, and there are several commercially available products that differ in their manufacturing process. Moreover, their use in pediatric dentistry has increased due to their favorable esthetics and mechanical properties [4,5]. Townsend et al. conducted a study to determine the maximum load forces required to fracture three types of all-zirconia crowns—EZ Pedo, NuSmile ZR, and Zirconia Kinder Krowns—and compared them to a control pre-veneered stainless-steel crown (NuSmile Signature crowns; NSW; NuSmile). They revealed that EZ Pedo zirconia crowns were significantly thicker in four out of six locations and thus had a greater fracture resistance than the other companies [6]. Another study investigated the fracture resistance of four commercially available primary anterior esthetic crowns and revealed that NuSmile zirconia crowns showed the highest load to fracture compared to Cheng Crowns [7]. Stawarczyk et al. (2019) compared the fracture resistance of three commercially available crowns with individually fabricated computer-aided design/computer-aided manufacturing (CAD/CAM) zirconia crowns, resin pre-veneered stainless-steel crowns, and conventional stainless-steel crowns. Their results showed that NuSmile had the highest fracture loads without pretreatment compared to other prefabricated brands, and after aging with saliva, Kinder Krowns showed the highest fracture loads compared to other prefabricated brands [8].

In recent years, prefabricated primary zirconia crowns for both anterior and posterior primary teeth have been developed [9]; however, there are limited studies regarding the mechanical properties of these crowns.

The purpose of this study was to evaluate the fracture resistance of three commercially available prefabricated zirconia crowns and their correlation with dimensional variance.

## 2. Materials and Methods

The study was carried out following institutional review board approval at the College of Dentistry at Prince Sattam bin Abdulaziz University (ID: PSAU2020025). Forty-two zirconia crowns were used from three companies: NuSmile primary zirconia crowns (NuSmile, Houston, TX, USA), Cheng Crowns Zirconia (Orthodontic Technologies Inc., Houston, TX), and Sprig EZ Crowns (Sprig, Loomis, CA, USA). The crowns were divided into 2 groups based on their location in the oral cavity (18 anterior/24 posterior) and then into subgroups based on the brand. The sizes used were (A4R) for NuSmile anterior (NA) and (E5L) for NuSmile posterior (NP), (CR4) for Cheng Crowns anterior (CA) and (ELL5) for Cheng Crowns Posterior (CP), (E4) for Sprig anterior (SA), and (K5) for Sprig Posterior (SP). The maximum dimensions of the samples were measured using an electronic dental caliper (length—*X*-axis, height—*Y*-axis, and width—*Z*-axis). Modeling wax (GEO Crowax and Perfect Wax) was used to create a negative replica of the crowns, which was later invested and turned into metal dies. The crowns were then tried out onto the corresponding metal dies to ensure a passive fit between each crown and metal die, which were then embedded into cylinders filled with orthodontic resin material for placement in the metallic holders. The crowns were cemented onto the dies with glass ionomer luting cement (Medicem, Neumuenster, Germany) following the manufacturer’s instructions and allowed to set for 24 h. A pressure of 30 Newtons was applied onto the crowns during cementation using a customized surveyor. The fracture resistance (N) was tested using a measured universal testing machine (INSTRON 5965. Software: Bluehill 3 version 3.22.1373, Norwood, MA, USA) with a 90° angle at a ramp rate of 1mm/min. The samples were placed into an appropriate metallic holder and the forces were applied onto the crowns with two different tips (chisel for anterior and flat rounded for posterior) until the crown fractured. The values were recorded at the time of fracture, and the Instron was reset after each value was recorded. Furthermore, the load was balanced, and the tip attached was returned to its starting position to ensure standardization.

Statistical analyses were performed using SPSS (IBM Corp. Released 2013. IBM SPSS Statistics for Macintosh, Version 22.0. IBM Corp., Armonk, NY, USA). Data were compared using 1-way analysis of variance at 95% confidence intervals (CI) and a significance level of 0.05 was used. Tukey’s post hoc test was used for inter-group comparisons. ANOVA was considered as there were more than two groups and the mean value for each group was compared. Data were explored for normality by checking the data distribution and using Kolmogorov-Smirnov and Shapiro-Wilk tests.

## 3. Results

**Figure 1:**
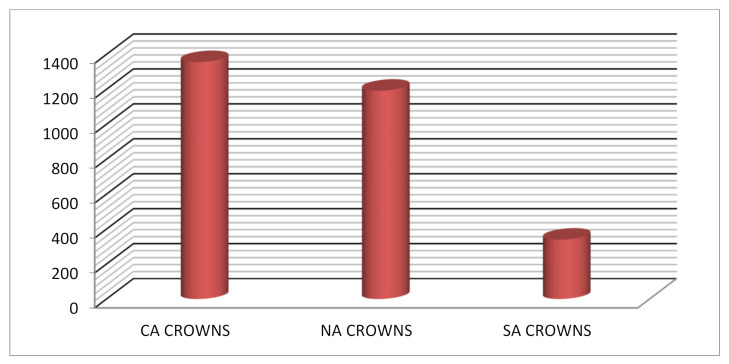
Shows mean load observed at anterior region among different types of crowns.

**Figure 2:**
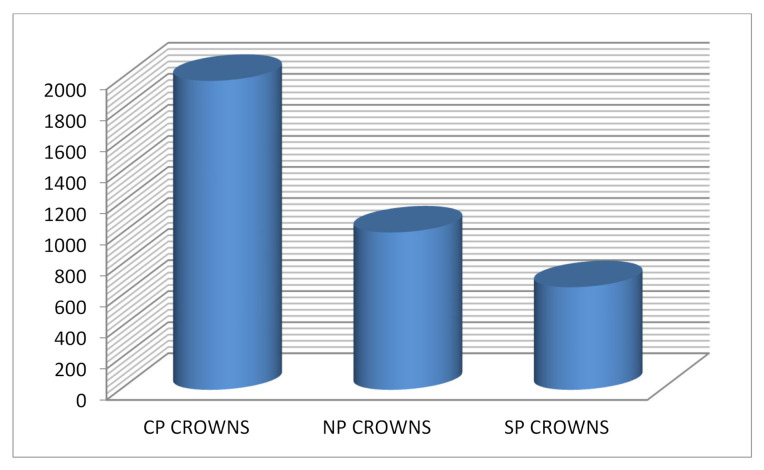
Shows mean load observed at posterior region among different types of crowns.

**Figure 3:**
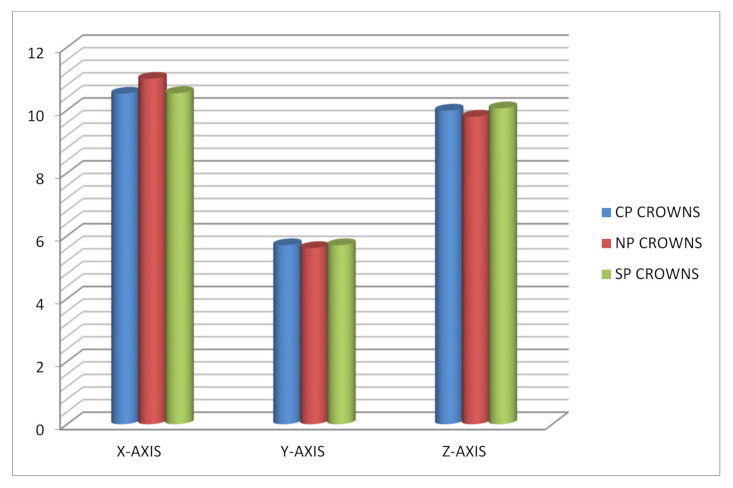
Shows mean fracture resistance observed with different types of posterior crowns at different axis.

A total of 42 crowns were tested (18 anterior crowns, and 24 posterior crowns). Failure was not noted in any of the samples; therefore, no samples were excluded. Table 1 shows the comparison of mean fracture resistance among three types of anterior and posterior crowns in three different dimensions (*x*-axis, *y*-axis, and *z*-axis); the total load was applied for the three different crowns. The mean load observed with the anterior crowns was highest with Cheng crowns (1355 ± 484), followed by NuSmile crowns (1192 ± 120). The least load was seen with Sprig crowns with a mean load of 339 ± 94. The mean load observed was statistically significant with an ANOVA value of 20.76 and a *p*-value of <0.001. The mean load observed with the posterior crowns was highest with Cheng crowns (1990.63 ± 485.39) followed by NuSmile crowns (1013 ± 240). The least load was seen with Sprig crowns with a mean load of 661 ± 184. The mean load observed was statistically significant with an ANOVA value of 34.77 and a *p*-value of < 0.001. Table 2 and Table 3 show the post hoc test with *p*-values among anterior crowns and posterior crowns along different axes. *p* < 0.05 was considered as statistically significant. The following Figure 1, Figure 2 and Figure 3 provide a clearer comparison between the different variables of dimensions and fracture load.

## 4. Discussion

Zirconia has emerged as an ideal material to use in fabricating crowns since it meets clinical requirements and has the ability to withstand masticatory forces [10,11,12,13,14,15]. In recent years, pediatric zirconia crowns have been widely used since they have proved promising alternatives to SSCs and pre-veneered crowns.

In the present study, Cheng’s zirconia crowns demonstrated the highest fracture resistance followed by NuSmile; the least fracture resistance was demonstrated by Sprig.

Townsend et al. (2014) measured the fracture resistance of three commercially available pediatric zirconia crown companies (Cheng Crowns, EzPedo (Sprig), and Kinder Krowns) and its correlation with crown thickness and used pre-veneered stainless-steel crowns as a control group. The findings concluded that the pre-veneered group was stronger than any of the zirconia groups and that there was a directly proportional relationship between an increase in both thickness and resistance to fracture. However, in this study, EZpedo (Sprig) zirconia crowns were more resistant to fracture than Cheng zirconia crowns [6]. Moreover, the study established a correlation between an increase in thickness of zirconia crowns and resistance to fracture—a result that also shares commonality with this study.

A similar study conducted by Al Shobber et al. (2017) compared the fracture resistance of two commercially available primary esthetic crown companies (NuSmile and Cheng Crowns) and two types of esthetic crowns (zirconia and pre-veneered stainless-steel crowns). Similarly to this study, the samples were tested for their fracture resistance using an Instron model 5965, with a 90° angle and ramp rate of 1mm/min, after the samples were cemented and mounted onto negative replicas. The results showed that Nusmile zirconia measured the highest fracture resistance (937 ± 131 N) followed by Cheng Crowns zirconia (751 ± 102 N); NuSmile pre-veneered (482 ± 76 N) and Cheng Crowns pre-veneered (415 ± 12 N) did not demonstrate a major difference. Overall, the study concluded that zirconia crowns were higher in fracture resistance compared to pre-veneered crowns, especially NuSmile crowns [7]. It is evident that the findings of the study are inconsistent with a previous study carried out by Al Shobber et al. (2017), where NuSmile zirconia crowns had a higher fracture resistance then Cheng zirconia crowns.

Stefan Kist et al. (2019) conducted a study in which prefabricated esthetic crowns were compared with conventional restorative crowns. They compared commercially available zirconia crowns, CAD/CAM-manufactured zirconia crowns, pre-veneered stainless-steel crowns, and conventional stainless-steel crowns under two variables which were fracture loaded under three conditions (no pretreatment, artificial aging, and chewing simulation/thermocycling). Pre-veneered stainless-steel crowns exhibited the highest fracture resistance (6251 N) within the no-treatment group, while CAD/CAM zirconia crowns had a fracture resistance of 2444 N and prefabricated zirconia crowns had a maximum fracture resistance of 1582 N. However, in the saliva aging and chewing simulation groups, the fracture resistance of pre-veneered stainless-steel crowns was negatively affected (5348 N for the aging group and 3778 N for the chewing simulation group) while the fracture resistance of the zirconia crowns remained similar. Furthermore, when gauging the survival rate, zirconia crowns and pre-veneered stainless-steel crowns had a 100% survival rate during chewing simulation, but only 41.7% in the conventional stainless-steel group [8].

Another study conducted by Beattie et al. (2011) evaluated the fracture resistance of three brands of primary esthetic stainless-steel crowns. The crowns were cemented onto idealized epoxy dies with GIC and the samples were then tested using a universal testing machine. Moreover, in order to replicate a cusp contact environment, a ball fixture was used to deliver the forces on the samples with a ramp rate of 1mm/min. Data collected from the study were analyzed using a one-way analysis of variance. The study concluded that forces needed to fracture any of the three brands of SSCs were quite similar to each other (1730 N ± 50 N, 1826 N ± 62 N, and 1671 N ± 68 N). In addition, the recorded forces also exceeded the natural forces produced by biting [16].

Furthermore, Singh et al. (2020) evaluated the effects of full-mouth rehabilitation on bite forces in dental pediatric patients in both primary and mixed dentition. Thirty subjects—comprising an almost even number of male and female participants—were included in this study, and the maximum bite force was voluntarily recorded for each participant before the start of dental treatment and one month after completion. The collected data were analyzed, considering variables that might affect the maximum bite force generated by a participant such as gender, age, height, weight, and extent of caries. The total mean maximum force of all of the participants before treatment was 167 N, in which the male subjects recorded a mean biting force of 175 N and the female subjects recorded a mean biting force of 166 N. Prior to the completion of dental treatment, the bite forces of the participants were once again measured, and a mean maximum bite force of 182 N was recorded [17].

When considering the above-mentioned results obtained by Singh et al. (2020), it is clear that all three commercially available crowns have optimum mechanical properties which can tolerate excessive masticatory forces.

## 5. Conclusions

From our study, there is a direct correlation between the increase in dimensions and the zirconia crowns’ ability to withstand a higher load/force. The overall dimensions of the crowns were shown to be somewhat correlated to the crown’s strength. Importantly, a potential reason for Sprig zirconia crowns demonstrating the lowest fracture resistance scores was because of a retentive feature within the crowns. Cheng’s zirconia crowns showed the highest fracture resistance (Newton), followed by Nu Smile and, lastly, Sprig. In general, the zirconia crowns (anterior and posterior) tested showed optimum mechanical properties to withstand the masticatory forces.

## Figures and Tables

**Table 1:** The mean fracture resistance among primary anterior, and posterior zirconia crowns.

Type of Crowns	Mean	SD	SE	Min	Max	95% CI
CA Crowns	1355	484	197	799	2192	847–1863
NA Crowns	1192	120	49	1046	1335	1065–1318
SA Crowns	339	94	38	252	501	240–439
CP Crowns	1990	485	171	1537	3019	1584–2396
NP Crowns	1013	240	84	679	1434	813–1214
SP Crowns	661	184	65	449	977	506–815

CA = Cheng anterior crowns; NA = NuSmile anterior crowns; SA = Sprig anterior crowns; CP = Cheng posterior crowns; NP = NuSmile posterior crowns; SP = Sprig posterior crowns.

**Table 2:** Post hoc test with *p*-values among anterior crowns at different axes.

	Crown	Compared with	Mean Difference	Std. Error	*p*-Value
*X*-axis	CA crowns	NA crowns	−0.36	0.01	<0.001 *
		SA crowns	0.20	0.01	<0.001 *
	NA crowns	SA crowns	0.56	0.01	<0.001 *
*Y*-axis	CA crowns	NA crowns	−0.55	0.03	<0.001 *
		SA crowns	0.08	0.03	<0.001 *
	NA crowns	SA crowns	0.64	0.03	<0.005 *
*Z*-axis	CA crowns	NA crowns	−0.48	0.02	<0.001 *
		SA crowns	−0.22	0.02	<0.001 *
	NA crowns	SA crowns	0.26	0.02	<0.001 *
Load (N)	CA crowns	NA crowns	163	169	<0.001 *
		SA crowns	1016	169	<0.001 *
	NA crowns	SA crowns	852	169	<0.001 *

* Mean difference is significant at *p* < 0.005 level.

**Table 3:** Post hoc test with *p*-values among posterior crowns at different axes.

	Crown	Compared with	Mean Difference	Std. Error	*p*-Value
*X*-axis	CP crowns	NP crownsSP crowns	−0.47	0.01	<0.001 *
			−0.02	0.01	<0.001 *
	NP crowns	SP crowns	0.45	0.01	0.44
*Y*-axis	CP crowns	NP crowns	0.10	0.01	<0.001 *
		SP crowns	0.01	0.01	<0.001 *
	NP crowns	SP crowns	−0.09	0.01	0.88
*Z*-axis	CP crowns	NP crowns	0.19	0.09	0.11
		SP crowns	−0.07	0.09	0.72
	NP crowns	SP crowns	−0.27	0.09	0.02
Load (N)	CP crowns	NP crowns	976	165	<0.001 *
		SP crowns	1329	165	<0.001 *
	NP crowns	SP crowns	352	165	0.11

* The mean difference is significant at *p* < 0.005 level.

## Data Availability

Data will be made available upon reasonable request to the authors.
